# THE ROLE OF THE NATIONAL REGISTER OF BIOLOGICAL AGENTS IN HEALTH PROTECTION OF EMPLOYEES EXPOSED TO BIOLOGICAL AGENTS USED INTENTIONALLY AT WORK IN POLAND

**DOI:** 10.13075/ijomeh.1896.02522

**Published:** 2025

**Authors:** Anna Kozajda, Emilia Miśkiewicz

**Affiliations:** Nofer Institute of Occupational Medicine, Department of Chemical Safety, Biological Safety Unit, ŁÓDŹ, Poland

**Keywords:** occupational exposure, biological agents, biohazards, work hygiene, work conditions, National Register of Biological Agents

## Abstract

**Objectives:**

This communication is aimed at outlining the role of the National Register of Biological Agents (NRoBA) in the system of working conditions supervision in Poland.

**Material and Methods:**

The paper was prepared based on a review of Polish legislation related to employee health protection, as well as scientific literature and recommendations of expert organizations regarding the intentional use of biological agents.

**Results:**

Polish law obliges employers to protect the health and safety of employees occupationally exposed to harmful agents. The State Sanitary Inspection and the State Labor Inspection supervise the employer's fulfillment of these obligations. Occupational exposure to biological agents may result from their intentional use (e.g., in the biotechnology industry or a scientific laboratory) or be related to their unintentional presence (e.g., in healthcare, sewage treatment plants, municipal waste management plants). Making a distinction between these 2 types of exposure is important for employers in relation to their legal obligations. In the case of using harmful biological agents for scientific, industrial or diagnostic purposes, the employer is obliged to notify the State Sanitary Inspection. Such notifications from employers are gathered in the NRoBA. Its aim is to support hygiene supervision over the intentional use of biological agents and to increase the employer's attention to the protection of the health of employees exposed to these agents. The International Labor Organization (ILO) in 2023 published *Technical Guidelines on Biological Hazards in the Working Environment*, in which it recommended increasing the capacity for epidemiological surveillance by creating networks or dedicated websites to collect and analyze adverse events in employees of research and development laboratories. The NRoBA complies with these guidelines but in order to use it in epidemiological studies, it should be re-digitalized to extend the range of collected data.

**Conclusions:**

The NRoBA in its present form has been used in Poland for almost 2 decades. Now it is time for re-digitalization to ensure its full compliance with the ILO recommendations and to use it in epidemiological studies.

## Highlights

- The National Register of Biological Agents (NRoBA) enhances the hygienic supervision at work.
- The NRoBA is consistent with the ILO recommendations on biological hazards at work.
- The range of information provided by employers to the NRoBA should be extended.

The Labor Code is an essential legal act regulating the area of employee safety and health protection in Poland [1]. The system of working conditions supervision is shaped by a series of legal acts developed based on 2 pillars, the first being occupational hygiene, and the second occupational medicine [2,3]. Within the framework of occupational hygiene, the main role is played by the employer, whose activities are supervised by 2 state inspections, i.e., the State Sanitary Inspection (Państwowa Inspekcja Sanitarna − PIS) [4] and the State Labor Inspection (Państwowa Inspekcja Pracy − PIP) [5]. The employer is legally obliged to provide employees with safe and hygienic working conditions [1,2]. Hiring >100 employees requires an establishment of an occupational health and safety service, which plays an advisory and control role in the workplace in the field of occupational health and safety [1,2]. Moreover, the law indicates detailed conditions for the protection of the safety and health of employees exposed to particular groups of harmful agents, along with occupational exposure limits for these agents [6–9].

In addition, occupational health and safety issues concerning the functioning of facilities with specific types of conducted activities, e.g., medical diagnostic laboratories, genetic engineering laboratories, or medical and veterinary waste disposal and storage companies, are controlled by sector-specific regulations [10–12]. The employer is obliged to provide preventive medical care for employees, including initial, periodic and control examinations (after a break from work lasting >30 days, caused by an illness), carried out by the occupational medicine service [1,3,13].

The tasks of the occupational medicine service include protecting the health of employees against the negative influence of harmful and burdensome agents present in the work environment and other unfavorable conditions directly related to the work performed [3]. Systematic monitoring of employees’ health is an active form of contributing to the improvement of working conditions by the employer, which reduces occupational risks and enables the identification of those elements of health that are causally related to working conditions [3]. Another task of the occupational medicine service is to inform employees about ways to prevent the adverse health effects [3]. In the case of a suspicion of an occupational disease [14], the patient is referred to an occupational medicine doctor competent to make a medical judgment. This document forms the basis for diagnosing an occupational disease by a territorially competent unit of the PIS [1,3,4,14].

The areas of occupational hygiene that are particularly important in relation to the tasks of occupational health services include the prevention and diagnosis of negative health effects in employees exposed to harmful biological agents present in the work environment. Issues related to the health protection of employees exposed to bacteria, fungi, viruses and endoparasites are regulated by Polish law, similar to the entire European Union [6,15]. Based on the applicable regulations, biological agents, as a group of harmful agents in the work environment, include fungi, bacteria and similar organisms, viruses, transmissible spongiform encephalopathies and endoparasites that can cause infections, allergies or poisoning [6]. These agents are a distinct group of occupational harmfulness, invisible and imperceptible to human senses, but highly unpredictable in terms of their consequences for human health [16].

Biological agents, whose effects on human health are well known, have been assigned by experts to 4 risk groups (RG 1–4), maintaining a gradation of the risk they cause. The lowest RG 1 includes agents that pose no risk to human health, while the highest RG 4 includes agents that cause serious diseases in humans, are highly infectious and there is no effective prevention or treatment for them [15,17]. Assigning individual biological agents to RG 1–4 facilitates the selection of risk control methods and preventive measures, but is not sufficient to ensure full protection of the health of exposed workers. In the field of occupational exposure to biological agents, certain challenges still remain in occupational hygiene, such as:

– the variety of agents that may occur in the work environment;
– the dynamics of quantitative and qualitative changes in time and space;
– methodological difficulties with microbiological identification (still encountered despite significant methodological progress);
– the lack of binding occupational exposure limits (so far, there are rather limited prospects for their rapid development);
– some agents posing a risk even when humans are exposed to very low concentrations;
– individual agents reproducing at different rates depending on many variables, including interactions between separate species/genera (e.g., toxin production, the presence of organic matter and water, ambient temperature);
– the probability of mutations in the genomes of individual agents (e.g., changes in the length of the incubation period or in the level of virulence); this concerns particularly fungi and viruses, but also other pathogens [16].

Occupational exposure to biological agents may be intentional or unintentional. Intentional use of a biological agent in the work process applies, e.g., to the biotechnology industry and scientific or diagnostic research. Unintentional exposure results from the presence in the work environment of a source of biological agents potentially harmful to humans, e.g., an infected person or animal, contaminated biological material, sewage and municipal waste [18,19]. The distinction between these 2 types of exposure is important from the point of view of occupational health. The unintentional nature of exposure means that various biological agents may be present in the work environment in a time- and space-variable quantitative and qualitative arrangement. This type of exposure causes difficulties in precisely assessing the occupational risk level. The higher the awareness of the threat, the stronger the vigilance in exposed people, which increases the effectiveness of preventive measures.

In the case of intentional exposure, both the exposed employees and their employer know the taxonomic affiliation and pathogenic properties of the agent used. This, on one hand, facilitates risk control and, on the other, can promote routine behavior. There is a confirmation that the key to success in the employee health protection is awareness of the risk resulting from exposure to a hazard that is invisible and imperceptible to human senses. Therefore, it is extremely important that the employer reliably implements the periodic occupational health and safety training for employees exposed to biological agents in the scope of health risks and prevention measures [2,6]. At the same time, the increased attention of the employer and the occupational health and safety service operating in the workplace [20] is considerably achieved as a result of the hygienic supervision of working conditions carried out by national inspections, i.e., the PIS and the PIP. The frequency of hygienic inspections of working conditions in companies varies within Poland, usually depending on the type and level of exposure, the number of employees, and the irregularities noted during routine inspections in terms of occupational health and safety requirements.

In the case of the use of harmful biological agents for scientific, industrial or diagnostic purposes, the employer is obliged to inform the territorially competent unit of the PIS. The information should consist of the company name and address, the type of economic activities, the organizational unit or workplace where the exposure occurs, contact data to the person responsible for occupational health and safety in the company, the result of the occupational risk assessment, including the name of the harmful biological agent and the risk group, the type of work performed and the period of exposure, the planned preventive measures, and the number of exposed employees.

In Poland, the law requires employers to provide the above-described information to the PIS:

– at least 30 days before the first use of a harmful biological agent from RG 2–4,
– whenever there are significant changes that influence the employee's safety and health at work,
– within 30 days after the company ceases its economic activity,
– immediately, in the case of any failure or accident that could have caused the release of a harmful biological agent classified to RG 2–4 [6].

It should be emphasized that Polish law obliges employers only to make the notification but does not require obtaining the consent from the competent authorities to intentionally use agents being human pathogens [6].

In accordance with other applicable legal acts [21,22], Poland uses the National Register of Biological Agents (NRoBA) [23]. The data provided by employers to the PIS are gathered in a database which fulfils 2 functions. Firstly, it improves the supervision of working conditions in companies where harmful biological agents are used intentionally. Secondly, it encourages both employers and the staff responsible for occupational health and safety to pay more attention to the issue of protecting the health of employees and securing intentionally used biological agents from being released into the environment.

As noted above, the obligation to provide information on the intentional use of harmful biological agents is a crucial preventive measure. However, the provisions of the applicable law in Poland oblige employers to provide information only in relation to the harmful biological agents classified to RG 2–4 [6]. The list of biological agents from RG 2–4, which forms an annex to the biological regulation [17], does not include all biological agents that may be harmful to humans. In the introductory notes to the classification of harmful biological agents, the legislator indicated that those biological agents which have not been classified to RG 2–4 in the list are not, by default, classified to RG 1 (agents safe for healthy people with a properly functioning immune system). This provision means that companies, deliberately using potentially harmful biological agents which have not been included in the list of RG 1–4 are not obliged to provide such information to the PIS.

It is difficult to estimate the exact number of companies that, due to such wording in the biological regulation, avoid entry into the NRoBA. Moreover, in Poland there is no legally binding interpretation of the term “intentional use.” As already described above, the employer is obliged to provide the PIS with information on the use of a harmful biological agent for scientific and research, diagnostic or industrial purposes.

In practice, this provision is interpreted as the use of a reference strain originating from a known source (a biobank) and having a safety data sheet (a specification) confirming taxonomic affiliation with a list of adverse properties for human's health. With such an interpretation, the obligation to provide information escapes the intentional use of biological agents whose species affiliation has been confirmed independently by employees of a company, e.g., a microbiological laboratory or one having such a unit in its structure. This situation may now occur increasingly often since genetic methods, enabling the reliable identification of biological agents to the species/strain/type level, have become widely used.

The special threat could be the import of dangerous pathogens from areas where they are endemic. The COVID-19 pandemic, announced by the WHO in 2020, as well as the currently observed increased incidence of infectious diseases (including pertussis, scarlet fever and monkeypox) in Poland, the EU and worldwide [24–28] show that harmful biological agents are unpredictable in terms of virulence and the adverse health effects in humans. This issue, until recently only obvious to public health experts, is currently noticed by governments and probably all citizens [29]. Workplaces increasingly often become the outbreaks of infectious diseases [30–32]. It seems extremely hard to completely prevent an infectious agent from being brought into the workplace by an infected employee who is unaware of it. However, it is possible to effectively prevent the opposite situation, i.e., bringing an infectious agent outside the workplace by an employee who acquired the infection while working with that agent [6,15].

In 2023, the International Labor Organization (ILO) published *Technical Guidelines on Biological Hazards in the Working Environment*, indicating that, in accordance with international and national regulations and procedures, and where necessary, exposure to specific biological hazards and their health effects should be reported to the relevant statutory authorities [33]. This recommendation is consistent with the considerations described above, regarding the principles in force in Poland for informing the competent unit of the PIS about the deliberate use of a harmful biological agent. The authority supervising hygienic conditions at work should be informed about each biological agent deliberately used in the work process, for which the occupational risk assessment has indicated that it may cause negative health effects in humans.

The ILO recommends increasing the capacity for epidemiological surveillance by creating networks or dedicated websites to collect and analyze accidents, injuries, infections or adverse events experienced by workers in research and development laboratories [32]. Such laboratories usually carry out research using biological agents which, although they may have negative effects on humans, are not covered by the obligation to report employee exposure to the competent authority.

Another challenge is the safety of employees occupationally exposed to pathogens during field work outside the laboratory, e.g., conducting research in the natural environment of the occurrence of etiological agents of zoonotic origin, such as caves or forests [34].

Summarizing the communication, it should be highlighted that this topic finally merits special attention. First of all, it is worth discussing who should provide information on the deliberate use of harmful biological agents and what data should be contained in the information provided by the employer to the PIS to fulfill its role in the employee protection system. No less important is the digitalization of the NRoBA to reduce administrative burden and to enable all interested parties to access their resources easily.
